# Polyphenolic Contents and Antioxidant Potential of Stem Bark Extracts from *Jatropha curcas* (Linn)

**DOI:** 10.3390/ijms12052958

**Published:** 2011-05-05

**Authors:** Osamuyimen O. Igbinosa, Isoken H. Igbinosa, Vincent N. Chigor, Olohirere E. Uzunuigbe, Sunday O. Oyedemi, Emmanuel E. Odjadjare, Anthony I. Okoh, Etinosa O. Igbinosa

**Affiliations:** 1 Drexel University Medical School, St. Peter’s University Hospital, New Brunswick, NJ 08901, USA; E-Mail: uyi80@yahoo.com; 2 Applied and Environmental Microbiology Research Group (AEMREG), Department of Biochemistry and Microbiology, University of Fort Hare, Private Bag X1314, Alice 5700, South Africa; E-Mails: ladyisk@yahoo.com (I.H.I.); vchigor@ufh.ac.za (V.N.C.); silvanusdemi@yahoo.com (S.O.O.); odj4real@yahoo.com (E.E.O.); aokoh@ufh.ac.za (A.I.O.); 3 Department of Microbiology and Biochemistry, Ambrose Alli University, Ekpoma, Edo State PMB 14, Nigeria; E-Mail: eghosa_uz@yahoo.com; 4 Department of Microbiology and Biotechnology, Western Delta University, Oghara, Delta State PMB 10, Nigeria

**Keywords:** *Jatropha curcas*, antioxidant activity, polyphenolics, free radicals, scavenging capacity

## Abstract

We assessed the polyphenolic contents and antioxidant potential of the aqueous, ethanol and methanol stem bark extracts of *Jatropha curcas*. The total phenol, flavonoids, flavonols and proanthocyanidin contents of the extracts were evaluated to determine their effect on the antioxidant property of this plant, using standard phytochemical methods. The antioxidant and free radical scavenging activity of ethanol, methanol and aqueous extracts of the plant were also assessed against 2,2-diphenyl-1-picrylhydrazyl (DPPH), 2,2′-azino-bis-(3-ethylbenzothiazoline-6-sulphonic acid) (ABTS), ferric reducing, nitric oxide (NO), superoxide anion, (O_2_^−^) and hydrogen peroxide (H_2_O_2_) using spectroscopic methods and results were compared with that of butylated hydroxyl toluene (BHT) and ascorbic acid as standards. The concentrations of different classes of phenolic compounds were higher in methanol and ethanol extracts compared to aqueous extracts. There was correlation between total phenol, total flavonoids, total flavonol and total proanthocyanidins (*r* = 0.996, 0.978, 0.908, and 0.985) respectively. There was correlations between the amount of phenolic compounds and percentage inhibition of DPPH radicals scavenging activity of the extract (*r* = 0.98). Findings from the present study indicated that *J. curcas* is a potential source of natural antioxidants and may be a good candidate for pharmaceutical plant based products.

## 1. Introduction

Oxidative stress, the consequence of the imbalance between prooxidants and antioxidants in an organism, is considered to play a very important role in the pathogenesis of several degenerative diseases [1]. These diseases include diabetes, aging, cancer, cardiovascular diseases, metabolic syndrome and atherosclerosis. Free radicals, such as hydroxyl, singlet oxygen, nitric oxide, hydrogen peroxide and superoxide radicals, are continuously generated in the cell, as a result of normal human metabolism. However, they can be harmful to the system if not properly regulated and thus may cause variety of pathological effect such as carcinogenesis, aging DNA damage and enzyme inactivation by attacking biological macromolecules. The mechanisms by which free radicals interfere with cellular functions are not yet fully understood, but one of the most important processes seems to be the formation of lipid hydroperoxides [1].

Polyphenolic substances possess many biological effects which are mainly attributed to their antioxidant activities in scavenging free radicals, inhibition of peroxidation and chelating transition metals [2]. For examples, flavonols, cinnamic acids, coumarins and caffeic acids are well known polyphenolic compounds with strong antioxidant properties. Hence play an important role in protecting food, cells and organs from oxidative damage [3]. These compounds (phenolic substances) all share the same chemical patterns, with one or more phenolic groups for hydrogen proton donors and neutralize free radicals [4–7].

Antioxidants can protect the human body from free radicals and reactive oxygen species (ROS) effects [8]. Antioxidant agents are well known to retard the progress of many chronic diseases as well as lipid peroxidation. Presently the most commonly used synthetic antioxidants are butylated hydroxyanisole (BHA), butylated hydroxytoluene (BHT), propylgallate and tert-butylhydroquinone. However, BHA and BHT have been restricted by legislative rules due to doubts over their toxic and carcinogenic effects [8,9].

*Jatropha curcas* (Linn) belonging to the family Euphorbiaceae is a shrub 4.5 to 8 m high. It has a smooth bark and milky latex. It is cultivated as an ornamental plant and live fencing at an altitude of 450–1300 m. The roots, stems, leaves, seeds and fruits of the plant have been widely used in traditional folk medicine in many parts of West Africa. The seeds have been used as purgative, anthelmintic and abortifacient, for treating ascites, gout and skin diseases [10]. Previous studies have reported the plant to exhibit such bioactive activities for fever, mouth infections, jaundice, guinea worm sores and joint rheumatism [11,12]. Fagbenro-Beyioku *et al*. [13] investigated and reported the anti-parasitic activity of sap and crushed leaves of *Jatropha curcas*. Mujumdar *et al*. [14] also reported that the crude methanolic extract from the root of *Jatropha curcas* exhibited anti-diarrhoeal activity in mice through inhibition of prostaglandin biosynthesis and reduction of osmotic pressure. Nevertheless, the plant has been shown to be a potential source of chemotherapeutic compounds [15]. Antimicrobial potentials and phytochemical properties of this plant have been reported elsewhere [16].

To the best of our knowledge there is paucity of information available in literature regarding the chemical composition and biological activity of *Jatropha curcas*. Therefore, this present study was designed to investigate total polyphenolic contents, antioxidant potential and free radical scavenging potentials of stem bark extracts of *Jatropha curcas* to understand the mechanisms of action underlying its ethnotherapeutic usage.

## 2. Results and Discussion

### 2.1. Polyphenol Content and Antioxidant Activity

The present study revealed relatively high level of total phenols, flavonoids, flavonols, and procanthocyanidins contents of the three solvents extract of *Jatropha curcas* as shown in Table 1. The concentrations of phenol (28.87 ± 1.04 mg/g tannic acid equivalent); flavonoids (11.18 ± 0.53 mg/g quercetin equivalents); Flavonols (12.55 ± 0.13 mg/g of quercetin equivalent) and proanthocyanidins contents (15.69 ± 1.86 mg/g of catechin equivalent) were high in methanolic extract while that of ethonolic extract was: phenol (14.47 ± 1.29 mg/g of tannic acid equivalent); flavonoids (9.33 ± 0.41 mg/g of quercetin equivalents), Flavonols (10.16 ± 0.29 mg/g of quercetin equivalent) and proanthocyanidins contents (12.33 ± 0.42 mg/g equivalent of catechin). The aqueous extract showed the least concentration of phenol (10.92 ± 2.25 mg/g of tannic acid equivalent), flavonoids (6.28 ±0.74 mg/g of quercetin equivalents), Flavonols (8.25 ± 0.17 mg/g of quercetin equivalent) and proanthocyanidins (7.74 ± 0.85 mg/g equivalent of catechin) contents. The concentrations of flavonoids were high in all the three solvents extract while phenols contents were very low when compared with other compounds. The differences in polarity of the antioxidant components are noticeably the reason why phenolic compounds and antioxidant activity of the extract differ. There was correlation between total phenols, total flavonoids, total flavonols and total proanthocyanidins (*r* = 0.996, 0.978, 0.908, and 0.985) respectively. The correlation coefficient between ABTS, DPPH assays and polyphenols is (*r* = 0.79, 0.88, and 0.98) respectively. Plant phenolics constitute one of the major groups of compounds acting as primary antioxidant or free radical terminators [5]. Synergism of polyphenolic compounds in the plant extract may contribute to its antioxidant activity [17]. The high antioxidant activity of the extracts could be attributed to the presence of phenolic compounds. The mechanism of actions of these compounds are unclear; however the observation could be due to the ability of phenolic compounds to absorb and neutralize free radicals, quench active oxygen species and decompose superoxide and hydroxyl radicals [18]. Flavonoids have been shown to exhibit their actions through effects on membrane permeability, and by inhibition of membrane-bound enzymes such as the ATPase and phospholipase A2 [19]. This property may explain the mechanisms of action of *J. curcas* and thus may be responsible for the ethnotherapeutic usage of this plant [20]. These observations support the usefulness of this plant in folklore remedies in the treatment of stress-related ailments as well as dressings for wounds normally encountered through bruises, and cuts [13].

### 2.2. DPPH Radical Scavenging Assay

**Figure 1.**
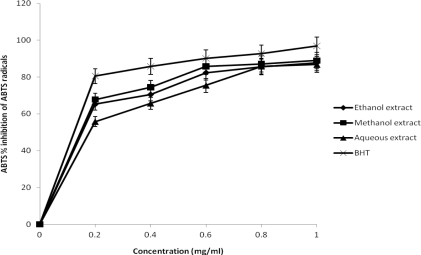
DPPH radical scavenging activities of the different solvents extract of *J. curcas*.

Free radicals and their scavenging activities play important role in the healing of normal and delayed healing types of wound [21,22]. DPPH scavenging activity assay has been utilized to gain the understanding of antioxidant potentials. The percentage inhibition DPPH scavenging activities of all the extracts were concentration dependent (Figure 1). The highest percent DPPH scavenging activity was shown by the methanolic extract (91.5%) followed by the aqueous extract (80.5%), whereas ethanolic extract exhibited the least value (78.2%). It was also observed that the scavenging activity of methanol extract was comparable with BHT (98.5%) used as standard drug at 1.0 mg/mL which was the highest concentration tested. The concentration-dependent curve of DPPH radical scavenging activity of *J. curcas* extracts compared well with BHT which suggests that the three solvent extracts possess high DPPH scavenging activity at the highest concentration (1.0 mg/mL). This is an indication that *J. curcas* can serve as a potential natural antioxidant over standard drugs. The findings of DPPH scavenging activity assay in this study indicates that the *J. curcas* was potently active. These results suggests that the plant extracts contain compounds that are capable of donating hydrogen to a free radical in order to remove abnormal electron which is responsible for radical’s reactivity. The ability of this plant to scavenge DPPH could also reflect its ability to inhibit the formation of ABTS^+^. These findings imply that, radical scavenging activity of the extract may be attributed to its strong proton donating ability.

### 2.3. ABTS Radical Scavenging Activity

**Figure 2.**
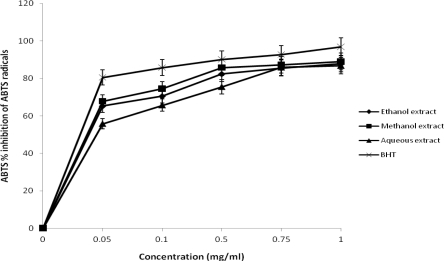
ABTS^+^ radical scavenging activities of the different extracts of *J. curcas*.

The percentage inhibition of ABTS radical by the plant extracts was concentration dependent. There was increased in ABTS radical scavenging activity with increasing concentration at different solvent extraction used in this study (Figure 2). At a concentration of 1.0 mg/mL, the percentage inhibition of methanol extract (89.0%); ethanol extract (87.78%) and aqueous extract (86.8%) showed similar trend but significantly different when compared with that of BHT (96.9%). High concentrations of the extract have been reported to be more effective in quenching free radical in the system [23]. The scavenging activity of ABTS^+^ by the plant extract was found to be remarkably high. The finding obtained in this study differ from previous study by Wang *et al*. [24] who reported that compounds which exhibit ABTS^+^ scavenging activity may not posses DPPH scavenging activity. The extracts were able to inhibit both DPPH and ABTS radicals with similar trend. This implies that the plant extracts could be useful for treating radical-related pathological damages especially at higher concentrations.

### 2.4. The Reducing Power of the Extract

**Figure 3.**
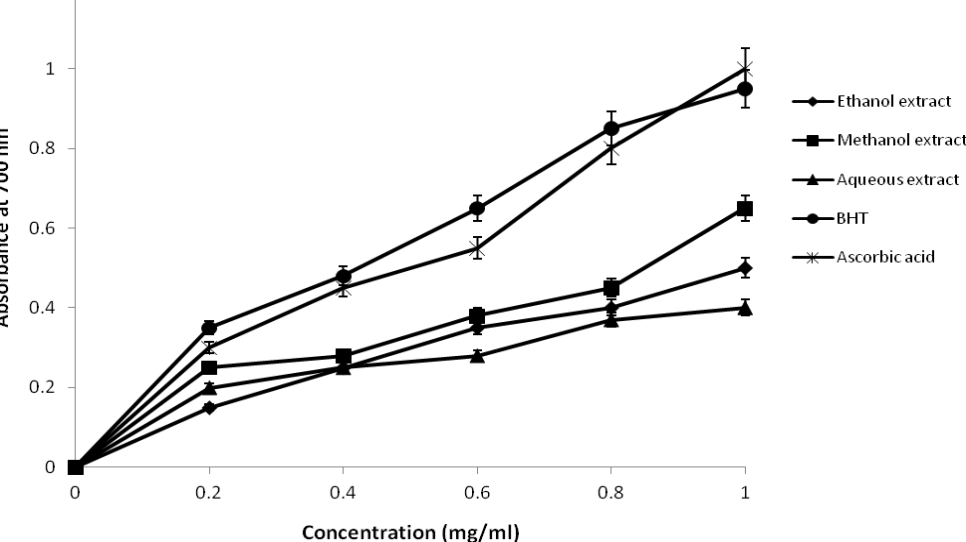
Total ferric reductive potential of the different solvents extract of *J. curcas*.

The reducing power of *J. curcas* extracts and the reference compounds increased with increasing concentration (Figure 3). The antioxidant potentials of the plant extracts was estimated from their ability to reduce Fe^3+^ to Fe^2+^. This was observed from yellow color of the test solution that changed to various shades of green and blue depending on the concentration of the plant extracts. The reducing value of the plant extracts was significantly lower than that of BHT and ascorbic acids, used as reference compounds in this study (Figure 3). There was significant difference in reducing activities between the plant extracts, BHT and ascorbic acids. However, reducing power of BHT and ascorbic acids was significantly higher (*P* < 0.01) than that of the plant extracts. The reducing power of the extracts, BHT and ascorbic acid increased with increasing concentration (Figure 3) in the following order, ascorbic acid > BHT > methanol > ethanol > aqueous extracts. Antioxidant activity has been shown to be related to the development of reductones, which are terminators of free radical chain reactors [5]. The presence of reductants such as antioxidant substances in the samples causes a reduction of the Fe^3+^ to Fe^2+^ form. Therefore, the ability of a compound to transfer electron is a significant indicator of its potential as an antioxidant [25]. The results showed that good correlations exist between reducing power, DPPH radical scavenging activity and total phenolics content of the extract. This indicates that the antioxidant compounds are electron donors and could reduce the oxidized intermediate of lipid peroxidation processes; thus acting as primary and secondary antioxidants [26,27].

### 2.5. Superoxide Anion Scavenging Activity

**Figure 4.**
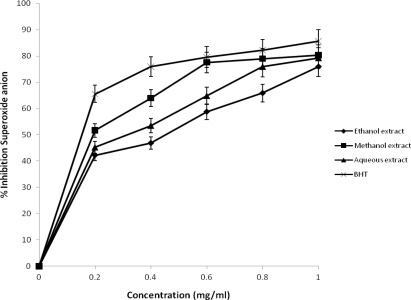
Percentage inhibition of superoxide anion radical scavenging activities of different extracts of *J. curcas*.

The percentage inhibition of superoxide anion by the extracts and standard drugs is shown in Figure 4. All solvents extracts of *J. curcas* have strong superoxide radical scavenging activity and exhibited higher superoxide radical scavenging activity when compared with BHT. The percentage inhibition of superoxide radical of BHT, methanol, aqueous and ethanol extracts of *J. curcas* at 1.0 mg/mL was found to be 85.69%, 80.29%, 79.23% and 75.92% respectively. All activity followed a concentration dependent manner and compared favorably well with the standard BHT. In the phenazine methosulphate/nicotinamide adenine dinucleotide-nitroblue tetrazolium (PMS/NADH-NBT) system, superoxide anion derived from dissolved oxygen by PMS/NADH coupling reaction reduces NBT. The decrease of absorbance at 560 nm with antioxidants thus indicates the consumption of superoxide anion in the reaction mixture. Superoxide anion radical is one of the strongest reactive oxygen species among the free radicals that are generated [28]. The scavenging activity of this radical by the plant extract suggests its potent scavenger of superoxide radical.

### 2.6. Nitric Oxide Scavenging Activity

**Figure 5.**
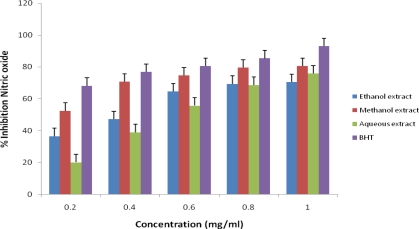
Percentage inhibition of nitric oxide scavenging activities of different extracts of *J. curcas*.

The extracts showed strong inhibitory activities with percentage inhibition of nitric oxide at 1.0 mg/mL the highest concentration of BHT, methanol, aqueous and ethanol extracts of *J. curcas* exhibit 92.95%, 80.50%, 75.89%, and 70.50% respectively, as shown in Figure 5. Nitric oxide is a reactive free radical produced from sodium nitroprusside in an aqueous solution at physiological pH and reacts with oxygen in the reaction to form nitrite. The extracts inhibit nitrite formation by directly competing with oxygen in the reaction with nitric oxide and other nitrogen oxides such as NO_3_, and N_2_O_3_ [29]. The level of nitric oxide was significantly (*P* < 0.01) decreased in this study by the plant extracts. The previous report on the anti-inflammatory role of nitric oxide [30] could support the use of *J. curcas* for treatment of inflammation and healing of wound.

### 2.7. Hydrogen Peroxide Scavenging Capacity

**Figure 6.**
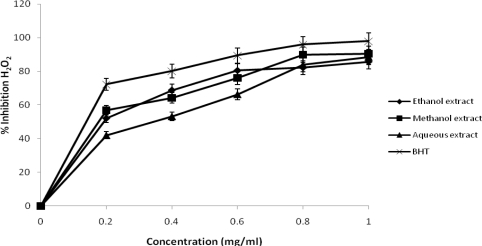
Percentage inhibition of hydrogen peroxide scavenging activities of different extracts of *J. curcas*.

The scavenging ability of aqueous, ethanol and methanol extracts (compared to BHT as standard) on hydrogen peroxide is shown in Figure 6. The percentage inhibition of hydrogen peroxide of BHT, methanol, aqueous and ethanol extracts of *J. curcas* at 0.8 and 1.0 mg/mL was found to be 95.82%, 89.93%, 83.92% and 82.19% respectively and 97.92%, 90.50%, 88.50% and 85.67% respectively. The extracts of *J. curcas* were capable of scavenging hydrogen peroxide in a concentration dependent manner and have a stronger hydrogen peroxide scavenging activity as compared with BHT. Hydrogen peroxide itself is not very reactive, but it could be toxic to cells because of its ability to penetrate biological membrance. As a result it may give rise to hydroxyl radical production in the cells [31]. Scavenging of H_2_O_2_ by the plant extracts may be attributed to their phenolics, which donate electron to H_2_O_2_, thus reducing it to water.

## 3. Material and Methods

### 3.1. Plant Materials

Fresh stem bark of *J. curcas* was collected from a local farm in Benin City, Edo State, Nigeria in the month of June, 2010 and was identified by the Botany Department of Ambrose Alli University, Ekpoma, Nigeria.

### 3.2. Chemicals

The chemicals used in this study include 2,2-diphenyl-1-picrylhydrazyl (DPPH), 2,2′-azino-bis-(3-ethylbenzothiazoline-6-sulphonic acid) (ABTS), butylated hydroxytoluene (BHT), 3-(2-pyridyl)-5,6-diphenyl-1,2,4-triazine-4′,4′-disulfonic acid, hydrogen peroxide, ferrous chloride, potassium ferricyanide, catechin, ascorbic acid, tannic acid, quercetin, nicotinamide adenine dinucleotide (NADH), trichloracetic acid (TCA), phosphate buffer, sulfanilic acid, glacial acetic acid, naphthylethylenediamine dichloride, folin-ciocalteu reagent, sodium carbonate, vanillin, aluminum chloride, ascorbic acid and potassium acetate were obtained from Sigma (Sigma-Aldrich GmbH, Sternheim, Germany). All other chemicals used, including the slovents were analytical grade.

### 3.3. Preparation of Extract

The fresh stem bark was air-dried to constant weight in the laboratory. The dried material was then pulverized using an electric blender (Pye Unicam, Cambridge, England) and stored in an air-tight container for further use. About 50 g of the pulverized plant material was extracted in 1000 mL of ethanol or methanol separately. Another 50 g of the powdered plant material was extracted in 1000 mL of cold distilled water maintained on a mechanical shaker (Stuart Scientific Orbital Shaker, Essex, UK) for 48 h. The separated extracts were then filtered through Whatman’s No. 1 filter paper. The ethanol and methanol filtrate were separately concentrated to dryness *in vacuo* using a rotary evaporator (Laborota 4000-efficient, Heldolph, Germany) to remove the solvents. The filtrate obtained from the aqueous was frozen at −40 °C and dried for 48 h using a freeze dryer Savant Refrigerated vapor Trap, (RVT 41404, CA, USA).

### 3.4. Determination of Total Phenolics Content

The total phenolics content of the extract were determined by Folin-Ciocalteu method described by Wolfe *et al*. [32] with little modification. To 0.1 mg/mL of the extract, was mixed 5.0 mL of 10% Folin-Ciocalteu reagent and 4.0 mL of sodium carbonate (75% w/v). The mixture was vortexed for 15 s and incubated at 40 °C for 30 min for color appearance. The absorbance was measured at 765 nm using spectrophotometer. Samples of the extract were evaluated at the final concentration of 0.1 mg/mL. The amount of total phenolic content was expressed as mg/g tannic acid equivalent using the expression obtained from the calibration curve: Y = 0.1216x, R^2^ = 0.936512, where x is the absorbance and Y is the tannic acid equivalent in mg/g.

### 3.5. Determination of Total Flavonoids Content

The total flavonoids were determined using the method of Ordonez *et al*. [27]. A volume of 0.5 mL of 2% AlCl_3_ ethanol solution was added to 0.5 mL of extract solution. The mixture was incubated for 1 h at room temperature for yellow color appearance; the absorbance was measured at 420 nm. Plant extracts were evaluated at a final concentration of 0.1 mg/mL. Total flavonoids content was calculated as quercetin equivalent (mg/g) using the equation obtained from the curve: Y = 0.255x, R^2^ = 0.9812, where x is the absorbance and Y is the quercetin equivalent.

### 3.6. Determination of Total Flavonols Content

The total flavonols content were determined using the method of Kumaran and Karunakaran [33]. Two milliliter (2.0 mL) of the sample was mixed with 2.0 mL of AlCl_3_ prepared in ethanol and 3.0 mL of 50 g/L sodium acetate solution were added. The mixture was incubated at 20 °C for 2.5 h after which the absorption was read at 440 nm using spectrophotometer. Plant extracts were evaluated at a final concentration of 0.1 mg/mL. Total flavonoids contents were calculated as quercetin (mg/g) using the following equation based on the calibration curve Y = 0.0255x, R^2^ = 0.9812, where x is the absorbance and Y is the quercetin equivalent.

### 3.7. Determination of Proanthocyanidins Content

The total proanthocyanidin were determined using the procedure reported by Sun *et al*. [34]. A volume of 0.5 mL of 0.1 mg/mL of extract solution was mixed with 3.0 mL of 4% vanillin-methanol solution and 1.5 mL hydrochloric acid, the mixture was allowed to stand for 15 min at room temperature, the absorbance was measured at 500 nm. Total proanthocyanidin contents were expressed as catechin (mg/g) using the following equation of the curve: Y= 0.5825x, R^2^ = 0.9277, where x is the absorbance and Y is the catechin equivalent.

### 3.8. 2,2-Diphenyl-1-picrylhydrazyl (DPPH) Radical Scavenging Assay

The method of Liyana-Pathiana and Shahidi [35] was used for the determination of scavenging activity of DPPH free radical in the extract solution. A solution of 0.135 mM DPPH in methanol was prepared and 1.0 mL of this solution was mixed with 1.0 mg of extract in methanol containing 0.2–1.0 mg/mL of the extract. The reaction mixture was vortexed thoroughly and left in the dark at room temperature for 30 min. The absorbance of the mixture was measured spectrophotometrically at 517 nm BHT was used as standard. The scavenging ability of the plant extract was calculated using this equation:

$$\text{DPPH}\;\text{Scavenging}\;\text{activity}\;(\%)=[({\text{Abs}}_{\text{control}}-{\text{Abs}}_{\text{sample}})]/({\text{Abs}}_{\text{control}})]\times 100$$

where Abs_control_ is the absorbance of DPPH + methanol; Abs_sample_ is the absorbance of DPPH radical + sample extract or standard.

### 3.9. 2,2′-Azino-bis-(3-ethylbenzothiazoline-6-sulphonic Acid) (ABTS) Radical Scavenging Assay

The method of Re *et al*. [36] was adopted for the determination of ABTS activity of the plant extracts. The stock solutions were of 7 mM ABTS^+^ and 2.4 mM potassium persulphate solutions. The working solution was then prepared by mixing the two stock solutions in equal quantities and allowing them to react for 12 h at room temperature in the dark. The solution was then diluted by mixing 1 mL ABTS^+^ solution with 60 mL of methanol to obtain an absorbance of 0.076 ± 0.001 units at 734 nm. Plant extracts (1 mL) at various concentrations 0.2–1.0 mg/mL of the extract, were allowed to react with 1 mL of ABTS^+^ solution, and the absorbance was measured at 734 nm after 7 min using spectrophotometer. The ABTS^+^ scavenging capacity of the extract was compared with that of BHT and percentage inhibition calculated as ABTS radical scavenging activity (%) = [(Abs_control_ − Abs_sample_)/(Abs_control_)] × 100 where Abs_control_ was the absorbance of ABTS^+^ radical + methanol; Abs_sample_ is the absorbance of ABTS^+^ radical + sample extract or BHT.

### 3.10. Determination of Ferric Reducing Power

The reducing power of the extracts was assayed according to the method of Duh *et al*. [18]. A volume of 1.0 mL of the extracts, BHT and ascorbic acid at different concentrations 0.2–1.0 mg/mL were mixed individually to the mixture containing 2.5 mL of 0.2 M phosphate buffer pH 6.6 and 2.5 mL potassium ferricyanide (K_3_Fe(CN)_6_) (1% w/v). The mixture was incubated at 50 °C for 20 min, followed by the addition of 2.5 mL of trichloroacetic acid (TCA) (10% w/v), centrifuged for 10 min at 1000 × g. The upper layer of the solution was collected and mixed with 2.5 mL of distilled water and 0.5 mL of ferrous chloride (0.1% w/v). The absorbance was measured at 700 nm in a spectrophotometer. The higher absorbance of the reaction mixture indicates strong reducing power of the plant extract.

### 3.11. Superoxide Anion Scavenging Capacity

Measurement of superoxide anion scavenging capacity of extracts was based on the method described by Liu *et al*. [28] with little modification. One millilitre (1.0 mL) of nitroblue tetrazolium (NBT) solution (156 mM NBT in 100 mM phosphate buffer, pH 7.4), 1.0 mL NADH solution (468 mM in 100 mM phosphate buffer (pH 7.4) and 100 μL of sample solution of extracts in distilled water and BHT were mixed individually at different concentrations 0.2–1.0 mg/mL. The reaction started by adding 100 μL of phenazine methosulphate (PMS) solution (60 mM PMS in 100 mM phosphate buffer, pH 7.4) to the mixture. The reaction mixture was incubated at 25 °C for 5 min, and the absorbance at 560 nm was measured. The percentage inhibition of superoxide anion was calculated using the following formula:

$$\%\;\text{Inhibition}\;\text{superoxide}\;\text{anion}\;\text{scavenging}\;\text{capacity}=[({\text{A}}_{\text{o}}-{\text{A}}_{1})/{\text{A}}_{\text{o}}]\times 100$$

where A_o_ is the absorbance of the control and A_1_ is the absorbance of extracts or standards.

### 3.12. Nitric Oxide Scavenging Activity

The method of Garrat [37] was used to determine the nitric oxide radical scavenging activity of extracts of *J. curcas*. A volume of 2.0 mL of mM sodium nitroprusside prepared in 0.5 mL phosphate buffer saline (pH 7.4) was mixed with 0.5 mL of plant extracts, BHT at concentrations (0.2–1.0 mg/mL). The mixture was incubated at 25 °C for 150 min. An aliquot of 0.5 mL of the solution was added to 0.5 mL of Griess reagents [(1.0 mL of sulfanilic acid reagent (0.33% prepared in 20% glacial acetic acid at room temperature for 5 min with 1.0 mL of naphthyethylenediamine chloride (0.1% w/v))]. The mixture was incubated at room temperature for 30 min. The absorbance was then measured at 540 nm. The amount of nitric oxide radical was calculated using the equation:

$$\%\;\text{Inhibition}\;\text{of}\;\text{nitric}\;\text{oxide}\;\text{radical}\;\text{scavenging}\;\text{activity}=[{\text{A}}_{\text{o}}-{\text{A}}_{1}]/{\text{A}}_{\text{o}}\times 100$$

where A_o_ is the absorbance of control and A_1_ is the absorbance of extracts or standards.

### 3.13. Hydrogen Peroxide Scavenging Capacity

The ability of the extracts to scavenge hydrogen peroxide was determined according to the method of Ruch *et al*. [38]. A solution of hydrogen peroxide (40 mM) was prepared in phosphate buffer (pH 7.4). Hydrogen peroxide concentration was determined spectrophotometrically measuring absorption with extinction coefficient for H_2_O_2_ of 81 M^−1^cm^−1^. Plant extracts (0.2–1.0 mg/mL) in distilled water was added to hydrogen peroxide solution (0.6 mL, 40 mM). Absorbance of hydrogen peroxide at 230 nm was determined after 10 min against blank solution containing the phosphate buffer without hydrogen peroxide. The percentage of hydrogen peroxide scavenging by the extracts and standard compounds was calculated following the equation:

$$\%\;\text{Inhibition}\;\text{Scavanged}\;[{\text{H}}_{2}{\text{O}}_{2}]=[({\text{A}}_{\text{o}}-{\text{A}}_{1})/{\text{A}}_{\text{o}}]\times 100$$

where A_o_ is the absorbance of the control, and A_1_ is the absorbance in the presence of the sample of extracts or standards.

### 3.14. Statistical Analysis

Experimental data were expressed as mean ± standard deviation (SD) of three replicates. Data were subjected to one-way analysis of varinace (ANOVA), and differences between samples were determined by Duncan’s multiple range test using the Statisitcal Analysis System (SAS version 8, SAS Institute, Cary, NC, USA). Correlation between polyphenol contents and antioxidant activity was ascertained by regression analysis.

## 4. Conclusions

The importance of polyphenolic bioactive components in the plant (*J. curcas*) extracts has been shown in this study. The presence of these bioactive components in *J. curcas* could be attributed to its pharmacological activities associated with free radicals. Although there could be several mechanisms of action of its effectiveness in free radical implicated diseases, the antioxidant and free radical scavenging properties of this plant seem to be highly significant. Consequently, the plant could play an important role in prevention of oxidative dependent diseases. Therefore, further investigations are needed for the isolation and identification of the active components and to elucidate its mechanisms of action as well as their potential in biological activity and antioxidant activities is ongoing.

## Figures and Tables

**Table 1.** Polyphenolic contents of the ethanol, methanol and aqueous extracts of the stem bark of *J. curcas*.

**Extracts**	**Total phenol (mg tannic acid/g)**	**Total flavonoids (mg quercetin/g)**	**Total flavonols (mg quercetin/g)**	**Total proanthocyanidins (mg catechin/g)**
**Ethanol**	14.47 ± 1.29	9.33 ± 0.41	10.16 ± 0.29	12.33 ± 0.42
**Methanol**	28.87 ± 1.04	11.18 ± 0.53	12.55 ± 0.13	15.69 ± 1.86
**Aqueous**	10.92 ± 2.25	6.28 ± 0.74	8.25 ± 0.17	7.74 ± 0.85

Note:

± = Standard derivation of triplicate.
